# 2-Deoxy-D-glucose Restore Glucocorticoid Sensitivity in Acute Lymphoblastic Leukemia via Modification of N-Linked Glycosylation in an Oxygen Tension-Independent Manner

**DOI:** 10.1155/2017/2487297

**Published:** 2017-07-26

**Authors:** Zaira Leni, Paulina Ćwiek, Valeriya Dimitrova, Andrea S. Dulcey, Nicola Zamboni, Cedric Simillion, Geetha Rossi, Kurt Leibundgut, Alexandre Arcaro

**Affiliations:** ^1^Division of Pediatric Hematology/Oncology, Bern University Hospital, Bern, Switzerland; ^2^Institute of Molecular Systems Biology, ETH Zürich, Zürich, Switzerland; ^3^Department of Clinical Research, University of Bern, Bern, Switzerland

## Abstract

In childhood acute lymphoblastic leukemia, treatment failure is associated with resistance to glucocorticoid agents. Resistance to this class of drugs represents one of the strongest indicators of poor clinical outcome. We show that leukemic cells, which are resistant to the glucocorticoid drug methylprednisolone, display a higher demand of glucose associated with a deregulation of metabolic pathways, in comparison to sensitive cells. Interestingly, a combinatorial treatment of glucocorticoid and the glucose analog 2-deoxy-D-glucose displayed a synergistic effect in methylprednisolone-resistant cells, in an oxygen tension-independent manner. Unlike solid tumors, where 2-deoxy-D-glucose promotes inhibition of glycolysis by hexokinase II exclusively under hypoxic conditions, we were able to show that the antileukemic effects of 2-deoxy-D-glucose are far more complex in leukemia. We demonstrate a hexokinase II-independent cell viability decrease and apoptosis induction of the glucose analog in leukemia. Additionally, due to the structural similarity of 2-deoxy-D-glucose with mannose, we could confirm that the mechanism by which 2-deoxy-D-glucose predominantly acts in leukemia is via modification in N-linked glycosylation, leading to endoplasmic reticulum stress and consequently induction of the unfolded protein response.

## 1. Introduction

Acute lymphoblastic leukemia (ALL) is the second most common cause of death in children and adolescents, behind accidents. Significant advances have been made in the successful treatment of childhood-ALL (ch-ALL), leading to an overall survival rate approaching 90% in children [1]. Despite this success, a subset of patients remains refractory to chemotherapy and suffers from relapse which is associated with poor outcome. The conventional ch-ALL treatment consists of several classes of chemotherapeutic agents including glucocorticoids (GCs) and fails in approximately 20% of the patients [2–4]. Resistance to GCs, such as methylprednisolone (MP), is related to unfavorable prognosis. Therefore, it is important to develop alternative therapies that can overcome MP resistance. Previous reports have suggested that leukemic cells from ALL patients resistant to GC treatment present an increase in glycolytic rate [5, 6]. Moreover, it has been shown that solid tumors have altered rates of glucose transport and glycolysis in comparison to their nonmalignant counterparts [7–10]. The treatment of ch-ALL with the glycolysis inhibitor 2-deoxy-D-glucose (2-DG) provided the first evidence that ALL have a similar metabolic shift as solid tumors [11]. 2-DG has been extensively described to competitively inhibit hexokinase (HK) and glucose phosphoisomerase (PGI) in solid tumors [12]. Indeed, 2-DG can enter into the cell through glucose transporter GLUT-1, where it is converted to 2-deoxy-D-glucose-6-phosphate (2-DG6P) by HK. Unlike the endogenous substrate, G6P, 2-DG6P cannot be further metabolized by the second enzyme in the glycolytic pathway, PGI. It has been shown that in solid tumors, 2-DG6P accumulation inhibits glycolysis which represents the main mechanism of 2-DG cytotoxicity [13]. Furthermore, it has been previously reported that in cancer cells growing under hypoxic conditions, 2-DG causes apoptosis by inhibiting glycolysis [12]. In this study, we demonstrate that, in contrast to solid tumors, 2-DG induces apoptosis in ch-ALL cell lines in combination with MP under normal oxygen tension. In addition, we report that the cytotoxic activity of 2-DG in leukemia cell line is not primary through glycolysis inhibition, since silencing of *HKII* does not interfere with 2-DG-mediated decrease in cell viability. We hypothesize that, in leukemia cells, 2-DG predominantly acts as an inhibitor of N-linked glycosylation (NLG) due to its structural similarity with mannose. 2-DG can be fraudulently incorporated in the place of mannose on the oligosaccharide chain and lead to accumulation of misfolded proteins [12]. The accumulation of misfolded proteins in the lumen of the endoplasmic reticulum (ER) leads to ER stress and, as a consequence, the induction of the unfolded protein response (UPR) [13]. Taken together, the cytotoxic activity of 2-DG in selected ch-ALL cells under aerobic conditions is caused by interfering with NLG rather than inhibition of glycolysis. Furthermore, we discovered an additional mechanism of 2-DG action, which involves glycogen synthase kinase-3 (GSK-3) in the context of MP resistance in ch-ALL cell lines.

## 2. Material and Methods

### 2.1. Calculation of Synergy

Synergistic effects of drugs were evaluated as equipotent drug concentrations by the equation developed by Berenbaum [14]. A dose-response curve was constructed for each single drug and combinations of two drugs together. The values calculated according to the Berenbaum equation are referred to as a synergy factor (*F*_syn_). A value < 1 indicates synergy, *F*_syn_ equal to 1 indicates an additive effect, and *F*_syn_ > 1 represents an antagonist effect.

### 2.2. Glucose-Consumption Assay

Glucose levels were measured with the glucose assay kit (Sigma, Buchs, Switzerland), as described by the manufacturer. Briefly, 5 × 10^5^ cells were cultured and after 96 h medium was collected and incubated for 30 minutes with glucose assay buffer (1.5 mM NAD, 1 mM ATP, 1 U/ml hexokinase and 1 U/ml glucose-6-phosphate dehydrogenase). During this time, glucose is phosphorylated to glucose-6-phosphate (G6P), and the latter is then oxidized to 6-phosphate glucoronate in the presence of NAD. Conversion of NAD to NADH was measured by the increase in absorbance at 340 nm, which is directly proportional to the glucose concentration. To calculate glucose consumption, values were compared with media (RPMI or IMDM) glucose levels and corrected for cell growth.

### 2.3. Flow Cytometry

Apoptosis was assessed by fluorescence-activated cell sorting (FACS) after treatment with MP and 2-DG or the combination of drugs. Cells were harvested, pelleted by centrifugation, and washed with phosphate-buffered saline (PBS). Subsequently, leukemic cells were resuspended in 100 *μ*l of binding buffer (1.4 M NaCl, 25 mM CaCl, 0.1 M Hepes; pH 7.4) and stained with FITC-labeled Annexin-V-fluorescein isothiocyanate (Biotium, Hayward, CA, USA) and 7-Aminoactinomycin D (7-AAD) according to the manufacturer's protocol (BD Biosciences, Mississauga, ON) and analyzed on a Becton-Dickinson LSR II flow cytometer using BD FACSDiva software (version 6.1.3; Becton Dickinson AG, Allschwil, Switzerland) and FlowJo software (version 5.4+; Three Star Inc., Ashland, OR, USA). PE Active Caspase-3 Apoptotic kit (BD Pharmigen, San Diego, CA, USA) according with manufacture's instruction was used to confirm apoptosis. Flow cytometry analysis measurements were performed in singlet with three repetitions of each individual experiment.

### 2.4. Nucleofection

Silencing of *HKII* gene by small interfering RNA (siRNA) was performed by nucleofection using the Amaxa Nucleofector II (Amaxa Biosystems, Cologne, Germany), following the manufacturer's instructions. Each sample was transfected with 300 nM siRNA (HKII: siRNA IDs 6562) (Ambion, Applied Biosystem, USA) using the program X-01 of the Nucleofector II. The transfection efficiency was evaluated by quantitative RT-PCR after 24 h. Following the same procedure, protein extracts were analyzed by Western blotting 48 h after transfection, and the impact on cell viability was evaluated by using the MTS assay (Promega, Madison, WI, USA).

### 2.5. Microarray Analysis

The raw data in the form of Affymetrix.CEL hybridisation files from the dataset of Haferlach et al. [15] were downloaded from the ArrayExpress database (accession E-GEOD-13159). The data was background-corrected and normalized using the Robust Microarray Analysis method. Gene summarization was done using the alternative CDF files provided by Dai et al. [16]. Differential gene expression of 121 ch-ALL samples carrying the translocation t(9;22) (Philadelphia chromosome positive) and 236 ch-ALL samples without this translocation compared to 72 healthy bone marrow samples was calculated using the limma R-package.

### 2.6. Metabolite Profiling

A total of 234 samples from SUP-15, SD1, and REH leukemia cell lines were treated with vehicle (Benzyl alcohol, AB), MP, and 2-DG or the combination for 0, 6, 12, 18, 24, and 48 h. Cells were extracted using a modified version of a previously described protocol. Briefly, rapid inactivation of metabolism was obtained by a two-step process consisting of initial quenching of the cells, followed by extraction of the metabolites. Quenching procedure: 2 × 10^6^ cells were washed twice with 150 *μ*l of ammonium carbonate (75 mM, pH 7.4) prewarmed at 75°C and shaken on a heating block. Cells were pelleted by centrifugation and snap-frozen in liquid nitrogen. For metabolite extraction, pellets were treated twice with 150 *μ*l of hot extraction solution (75°C, 70% *v*/*v* pure ethanol) and placed in a heating block (75°C) for 3 minutes. The collected supernatants were completely dried under vacuum. Dry extracts were stored at −80°C until metabolomics analysis. Nontargeted metabolome profiling was done by flow injection—time-of-flight analysis in negative mode on an Agilent 6550 QTOF instrument (Agilent, Santa Clara CA, USA).

### 2.7. Clustering Analysis and Metabolite Pathway Analysis

The ion intensities were first log-transformed and then averaged over all technical replicates per condition, resulting in a matrix with 60 columns for 4 treatments, 3 cell lines and 5 time points, and 827 rows for as many ions. For each noncontrol condition—that is, any treatment with MP, 2-DG, or 2-DG+MP—log-fold change values were calculated by subtracting the log-transformed intensities from the corresponding, that is, same cell line and time point, control sample. This operation resulted in a matrix with 45 columns—3 cell lines x 3 treatments x 5 time points—and 827 rows. Of these 827 ions, we retained only those with significantly changing intensities, either over time or across different treatments compared to the control treatment, using the limma method [17]. Using an adjusted *p* value cutoff of 0.01, 649 ions were retained. Next, k-medoids clustering was performed to detect groups of similarly regulated ions using the correlation distance as distance metric. Using the silhouette width quality score, the optimal number of clusters to create was found to be 5. We then used the SetRank algorithm to look for overrepresented pathways in each cluster, using metabolite annotation from the KEGG and Reactome databases [18, 19].

### 2.8. High-Throughput Protein Kinase Inhibitors Screen

REH and SD1 leukemia cells were screened with a library of 365 GlaxoSmithKline Published Kinase Inhibitor Set (GSK, PLC United Kingdom) [20]. The cells were plated at a minimum density of 30,000 cells per well, in 96-well plates in triplicates. Cells were treated with MP (150 *μ*g/ml for REH and 100 *μ*g/ml for SD1) alone and/or in combination with the kinase inhibitors at the final concentration of 1 *μ*M. Dimethyl sulfoxide (DMSO, Sigma-Aldrich Chemie Gmbh, Buchs, Switzerland) as a negative control was added to each plate of the library. Validation of the hit candidates was evaluated after 72 h using the MTS assay. SB-360741 a 3-anilino-4-arylmalemide, GSK-3 inhibitor was selected and further validated in SD1 and REH cell lines.

### 2.9. Statistical Analysis

All statistics were performed in GraphPad Prism software (La Jolla, CA, USA). The statistical significance of differences between groups was assessed by two-way analysis of variance (ANOVA) correct for Bonferroni's multiple comparison test. *p* values of <0.05 were considered significant.

## 3. Results

### 3.1. 2-DG Modulates MP-Induced Cytotoxicity in Resistant ch-ALL Cell Lines

**Figure 1:**
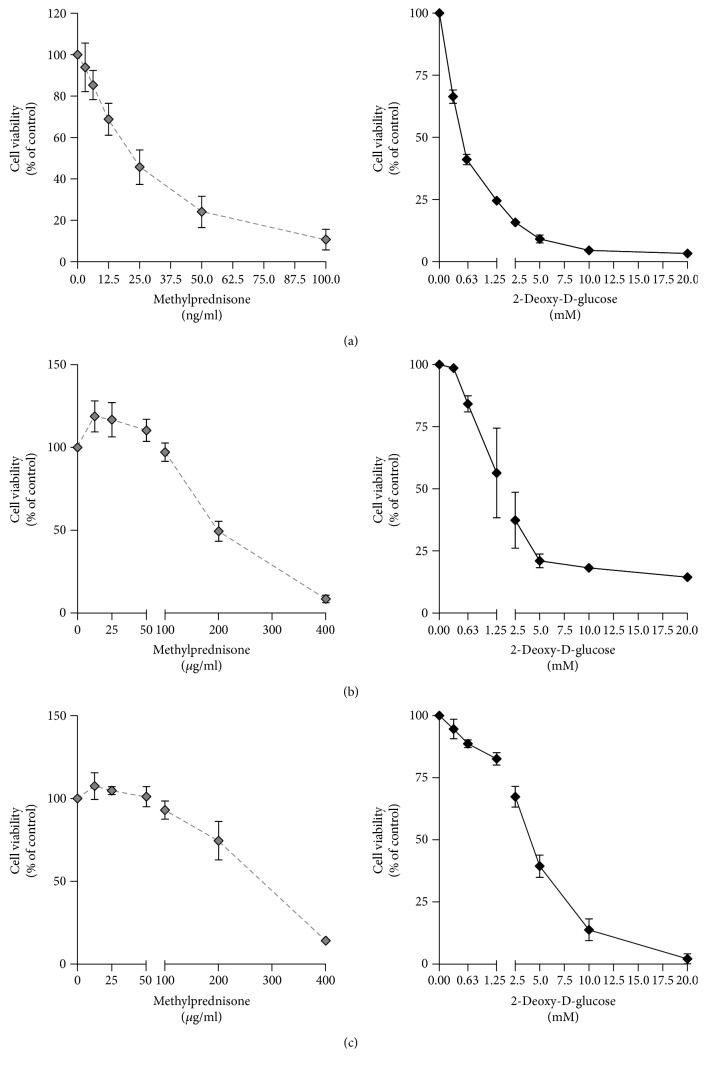
Effects of MP and 2-DG single treatment in a panel of ch-ALL cell lines. Cell viability was evaluated in (a) SUP-B15, (b) SD1, and (c) REH cells treated for 72 h with MP and 2-DG at the indicated concentrations. Error bars represent the SD of the mean of three independent experiments (*n* = 3).

**Figure 2:**
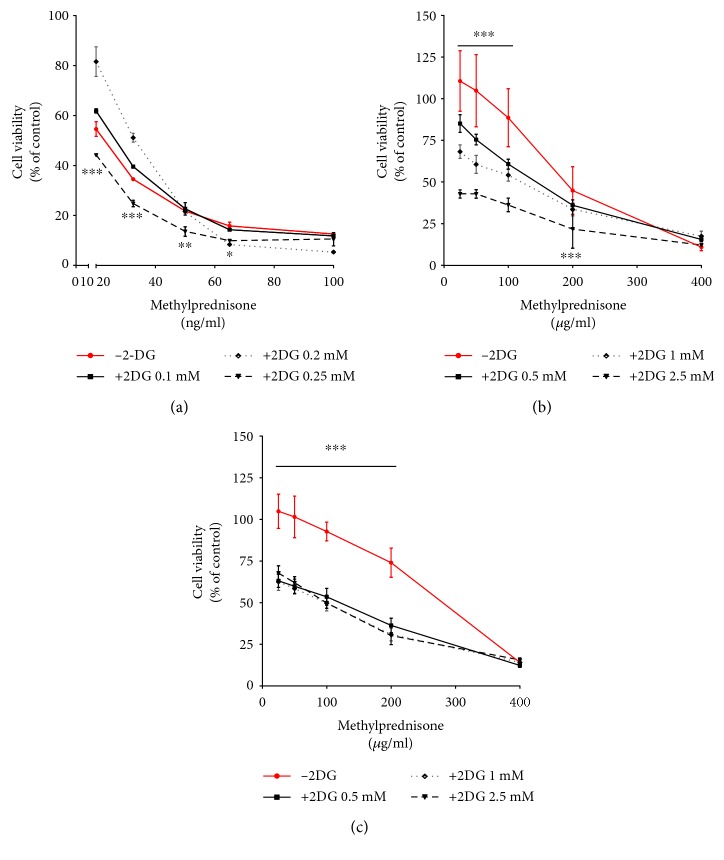
Synergistic effect of MP and 2-DG in ch-ALL cell lines. The responses of a panel of leukemia cell lines to MP alone and in combination with 2-DG: (a) 0.1 mM, 0.2 mM, and 0.25 mM of 2-DG for SUP-B15 and (b, c) 0.5 mM, 1 mM, and 2.5 mM of 2-DG for SD1 and REH was measured after 72 h using the MTS assay. Cell viability was normalized to untreated cells (100%). Data represent the SD of the mean of three independent experiments (*n* = 3). ^∗^*p* ≤ 0.05, ^∗∗^*p* ≤ 0.01, ^∗∗∗^*p* ≤ 0.001 compared to single treatment (−2-DG) as determined by two-way ANOVA analysis of variance using Bonferroni's multiple comparison test.

2-DG, a synthetic glucose analog with a primary function to inhibit glycolysis, has been reported to synergize with standard anticancer treatments in the induction of cell death in several carcinoma types [10, 21, 22]. MP resistance in leukemia has been associated with an increased expression of genes involved in glucose metabolism. Moreover, inhibition of glycolysis was shown to sensitize leukemia cells to prednisone [5]. Single treatment with MP was administrated to three leukemia cell lines, and according to their responses, they were classified as sensitive (SUP-B15), intermediate (SD1), and resistant (REH) cells toward MP (Figure 1). To determine the effects of 2-DG, SUP-B15, SD1, and REH, cells were exposed to increasing concentration of the glucose analog and their growth and cytotoxic responses were assessed (Figure 1). SUP-B15 and SD1 displayed a dose-dependent decrease in cell viability (Figures 1(a) and 1(b)) in response to the glycolysis inhibitor. The REH cell line showed a higher resistance towards 2-DG (Figure 1(c)). Thus, it was necessary to increase the dose of the glucose analog in order to obtain the same biological effect. To further investigate if the observed synergism of chemotherapy and 2-DG reported in carcinoma cells could also be observed in the case of GCs sensitization towards MP, 2-DG was administrated with sublethal concentrations of MP. Coincubation of 2-DG and MP displayed increased cytotoxicity in all leukemia cell lines tested. All three leukemia cell lines displayed synergistic effects between the two drugs, with values of *F*_syn_ = 0.86, 0.95, and 0.58 for SUP-B15, SD1, and REH, respectively (Figure 2). Moreover, SD1 displayed a dose-dependent viability decrease upon combinatorial treatment with all three concentrations of 2-DG tested (Figure 2(b)), while the effect on REH cell line was independent of the dose of 2-DG (Figure 2(c)). In contrast, the synergistic effect in SUP-B15 was observed only at the higher concentration of 2-DG (0.25 mM) (Figure 2(a)).

### 3.2. 2-DG Inhibits Glucose Consumption, Utilization, and Uptake

**Figure 3:**
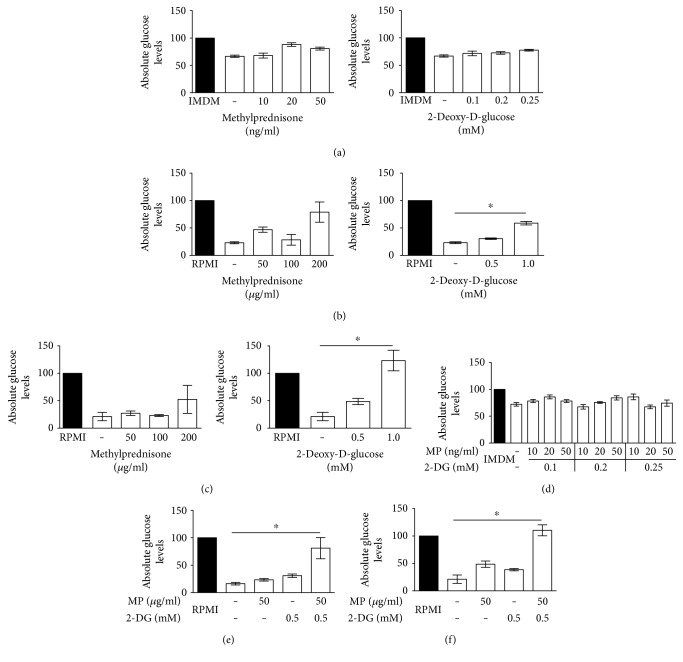
Glucose consumption in a panel of ch-ALL cell lines. The absolute glucose levels were measured in the cell media after incubation with MP, 2-DG, or combinatorial treatment. Kinetic measurements of glucose uptake were performed; only data from 96 h are shown. (a) SUP-B15, (b) SD1, and (c) REH cell lines were treated with increasing concentration of MP (left graph) and 2-DG (right graph). RPMI and IMDM controls represent the amount of glucose present in the culture medium incubated for 96 h in the absence of treatment. The effects of combined drug treatments on glucose levels are shown in (d), (e), and (f) for SUP-B15, SD1, and REH, respectively. The conversion of glucose to glucose-6-phosphoglucoronate (G6P) and NADH was evaluated. The concentration of NADH was measured. Graphs represent the SD of the mean of at least two independent experiments (*n* = 2). ^∗^*p* ≤ 0.05 using two-way analysis of variance (ANOVA) using Bonferroni's multiple comparison test.

To determine whether metabolic changes are associated with glucocorticoid resistance in ch-ALL and to further investigate the hypothesis that this cancer relies on glycolysis to produce ATP and biomass, we analyzed the glucose uptake in a panel of leukemia cell lines (Figure 3). The cells were cultured in normal media in the presence or absence of single drugs or combinatorial drug treatments. Despite the broad spectrum of treatment concentrations, the SUP-B15 cell line did not display any differences in glucose uptake upon treatment with MP (Figure 3(a)), 2-DG (Figure 3(b)), or the combination suggesting that the survival of this cell line does not rely exclusively on glycolysis (Figure 3(d)). This observation can be in part explained by SUP-B15 sensitivity to MP. In contrast, SD1 and REH cells displayed an increase in glucose consumption and a significant rescue of the glucose uptake upon 2-DG treatment (Figures 3(b) and 3(c)) at the concentration of 1 mM. Moreover, the combinatorial treatment of MP (50 *μ*g/ml) and 2-DG (0.5 mM) was able to revert the glucose uptake significantly, after 96 h exposure (Figures 3(e) and 3(f)).

### 3.3. 2-DG Sensitizes Resistant ch-ALL Cell Lines to MP-Induced Apoptosis

**Figure 4:**
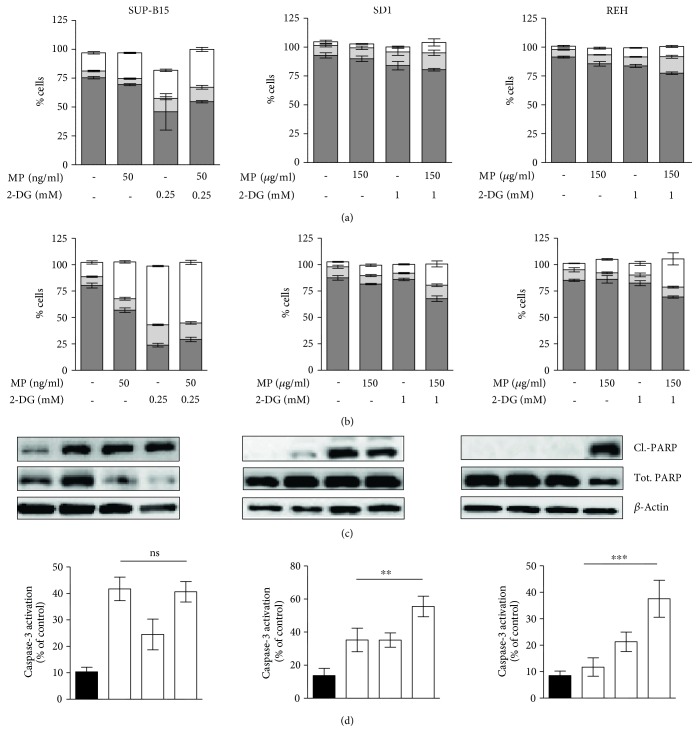
Induction of apoptosis upon MP, 2-DG, and combinatorial treatments. Apoptotic markers analysed by fluorescence-activated-cell-sorting (FACS) and western blot in a panel of ch-ALL after treatment with MP and 2-DG. FACS quantification of single (light grey) or double positive cells for Annexin-V and 7-AAD (white) after (a) 12 h and (b) 24 h treatment with MP and 2-DG. The percentage of living cells is represented in dark grey color. (c) Protein expression analysis by Western blot of cleaved-PARP in leukemia cell lines treated with MP, 2-DG, and MP+2-DG for 24 h is shown. (d) Caspase-3 activation was assessed by FACS in all three leukemia cell lines treated as described previously. Error bars represent the SD of the mean of at least two independent experiments (*n* = 2). ^∗∗^*p* ≤ 0.01, ^∗∗∗^*p* ≤ 0.001, two-way analysis of variance (ANOVA) using Bonferroni's multiple comparison test.

To analyze the mechanism of action of the combinatorial treatment of 2-DG and MP as a cause of cell viability decrease, a flow cytometry analysis of apoptotic markers (Annexin-V, 7-AAD) was performed. Induction of apoptosis was detected in all three leukemia cell lines after 12 h and 24 h (Figures 4(a) and 4(b)). An increase in early apoptosis (Annexin-V-positive cells) was observed after 12 h in the intermediate and resistant cell lines (Figure 4(a)). Unlike SD1 and REH, SUP-B15 displayed an increase in late apoptosis upon 12 h of treatment. Interestingly, combinatorial treatments induced early and late apoptosis in all three leukemia cell lines (Figure 4(b)). Taken together, these results confirm that MP treatment in combination with 2-DG not only leads to increased apoptotic activity in leukemia cell lines, but also tends to overcome MP-resistance in SD1 and REH by inducing a decrease in cell viability. This supports the hypothesis that inhibition of metabolism with 2-DG impairs proliferation and triggers cell death in ch-ALL cells. To confirm our data, we measured apoptotic markers upon single and combinatorial treatment at the protein level. An induction of apoptosis was observed in all three leukemia cell lines upon 24 h of treatment, indicated by the increased levels of cleaved PARP protein, a nuclear enzyme involved in DNA repair (Figure 4(c)). SUP-B15 displayed a similar induction of cleaved PARP upon single or combinatorial treatment while SD1 demonstrated an induction of apoptosis upon 2-DG treatment and the combinatorial treatment (Figure 4(c)). REH displayed apoptosis only following combinatorial treatment (Figure 4(c)). Moreover, the levels of active caspase-3 were assessed after 24 h of treatment. In line with our previous results, activation of caspase-3 in the SUP-B15 cell line was found upon all treatment conditions, while in SD1 and REH, a significant activation was observed only upon combinatorial treatment (Figure 4(d)). Regression analysis confirmed that the reduction in cell viability is in part due to the induction of apoptosis (Additional file 1: Figure S1 available online at https://doi.org/10.1155/2017/2487297).

### 3.4. 2-DG-Induced Cytotoxicity Is Exerted in a Hexokinase-Independent (HK) Manner in Leukemia Cell Lines

**Figure 5:**
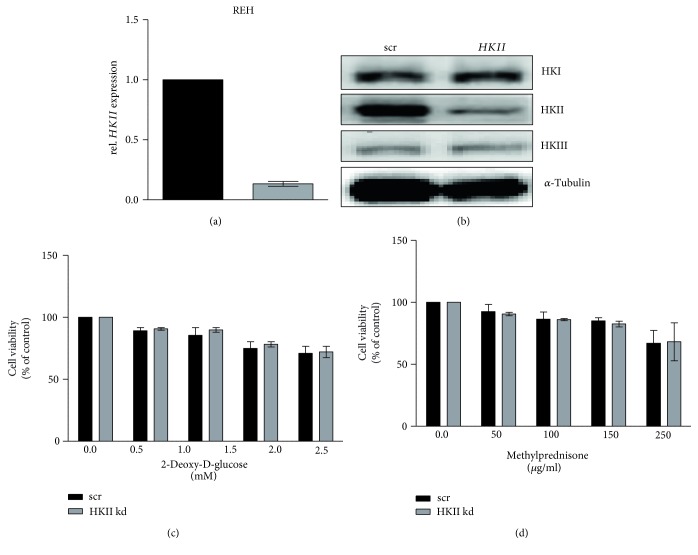
Gene expression analysis. REH transfection with siRNA was performed using nucleofection and gene expression was assessed. (a) Expression of HKII after silencing was measured in REH cells line at the mRNA level (24 h) and (b) at the protein level (48 h). The effects of (c) 2-DG or (d) MP on cell viability were measured in the presence or absence of HKII expression. The fold change was calculated via normalization to the scrambled control. Error bars represent the SD of the mean of at least three independent experiments (*n* = 3).

We used the cDNA microarray data set from Haferlach et al. [15] to analyze differences in gene expression in patients of two ch-ALL subgroups in comparison with healthy bone marrow samples. The wide cohort of ch-ALL patient samples analyzed in our laboratory (data not shown) displayed a general downregulation of the genes involved in the glycolytic pathway (Additional file 1: Figures 2a and 2b). Surprisingly, all three hexokinase isoforms (HKI, HKII, and HKIII) are significantly downregulated in both ch-ALL subgroups examined compare to healthy bone marrow (all *p* value >10^−10^) (Additional file 1: Figure 2a). The hexokinase isoforms II (HKII) and III (HKIII) are enzymes responsible for the conversion of glucose to glucose-6-phosphate (G6P), which is considered as the first limiting step of glycolysis. In line with this result, further evidence revealed that in our panel of leukemia cell lines, the expression of HK isoforms was downregulated (Additional file 1: Figure 2c). We thus speculated that in our leukemia in vitro model, HKII is not required for 2-DG-mediated apoptosis induction. Since leukemic cell can circulate freely through normoxic and hypoxic environments, we investigated whether in normal and decreasing oxygen tension, 2-DG leads to the same biological effect. Upon treatment with 2-DG, the cells were incubated either in normoxia or in hypoxia (pO2 1%). No significant differences in the impact of 2-DG on cell viability were observed (Additional file 1: Figure 3). Thus, 2-DG treatment leads to cell viability impairment independently of the oxygen levels in ch-ALL cells. To confirm that HK is not crucial for the effects of 2-DG in leukemia cell lines, we performed HKII silencing in all three leukemia cell lines (Figure 5 and Additional file 1: Figure 4). Due to low transfection efficiency in SUP-B15 and SD1 cell lines and since REH appeared to be the most resistant cell line, further experiments were carried out only in REH. The siRNA transfection efficiency at the mRNA level was measured after 24 h (Figure 5(a) and Additional file 1: Figure 4b) and confirmation of HKII silencing at the protein level was measured after 48 h by Western blot analysis (Figures 5(a) and 5(b)). To gain further insight on the role of HKII in REH cells, we performed cell viability assays after HKII knockdown. Silencing HKII did not significantly change the treatment response to either 2-DG (Figure 5(c)) or MP (Figure 5(d)), implying that the cytotoxic effect of 2-DG in this ch-ALL cell line is not dependent on its ability to inhibit glycolysis. Therefore, we focused our attention on identifying additional molecular targets of 2-DG in leukemia cell lines.

### 3.5. 2-DG Interferes with N-Linked Glycosylation (NLG) and Induces Changes in the Metabolic Profile of Leukemic Cells In Vitro

**Figure 6:**
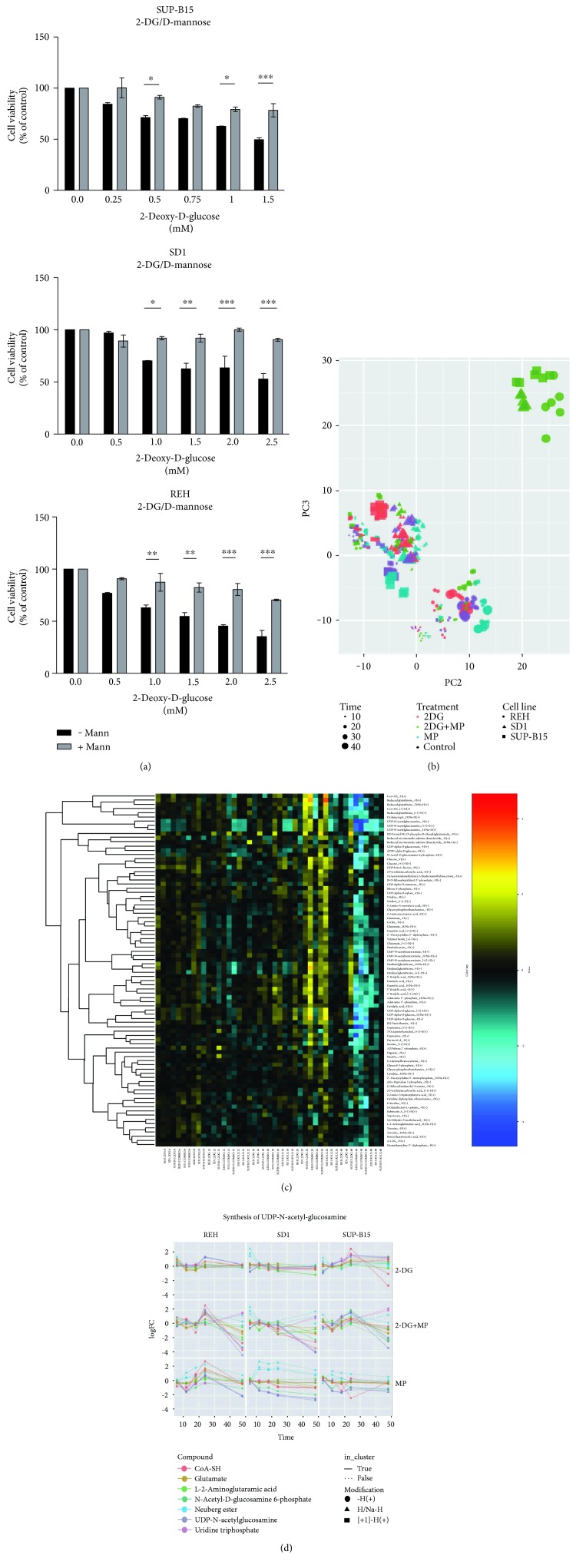
Metabolic profiles upon treatment with 2-DG and MP in ch-ALL. (a) Reversal effects of 2-DG on cell viability by exogenous mannose (2.5 mM). (b) Principal component analysis (PCA) score plot of SUP-B15 (●), SD1 (▲), and REH (■). Representative data are shown; the size of each condition is proportional to the treatment exposure. (c) The hierarchical clustering heat map shows consistent metabolic changes of ch-ALL cell lines upon 48 h of combinatorial treatment. (d) Enrichment in metabolites involves in the synthesis of the UDP-N-acetyl-glucosamine pathway in a matrix of 60 columns for 4 treatments (ctrl, 2-DG, MP, and 2-DG+MP) and 5 time points (0, 6, 12, 18, 24, and 48 h). Error bars represent the SD of the mean of at least three independent experiments (*n* = 3). ^∗^*p* ≤ 0.05, ^∗∗^*p* ≤ 0.01, ^∗∗∗^*p* ≤ 0.001, two-way analysis of variance (ANOVA) using Bonferroni's multiple comparison test.

2-DG is known as a structural analog of mannose and acts as a potent inhibitor of N-linked glycosylation (NLG), by competition with the endogenous substrate and by fraudulent incorporation into dolichol-pyrophosphate (lipid)-linked oligosaccharides, which are the precursors of NLG [23]. To investigate whether the antiproliferative effects of 2-DG in normoxia predominantly occur through inhibition of NLG and not through an HK-dependent block in glycolysis, we performed a cytotoxicity assay in the presence or absence of mannose (2.5 mM) (Figure 6(a)). Mannose treatment significantly reversed 2-DG-induced cytotoxicity, leading to the hypothesis that the effects of 2-DG in leukemia cells in normoxia are primarily due to interference with NLG. Moreover, a metabolomics analysis of the leukemia cell lines under study showed that a significant decrease in several metabolites involved in hexosamine biosynthesis pathway (HBP) and in the genesis of N-linked glycans was observed upon 48 h of combinatorial treatment (Figure 6(b)). UDP-N-acetyl-hexosamine, N-acetyl-hexosamine-phosphates, and UDP-hexosamine, which are all involved in the generation of glycosaminoglycans, proteoglycans, and glycolipids, were significantly downregulated upon combinatorial treatment (all *p* value <10^−15^) (Figure 6(c)). As a consequence, the glycans are not transferred to the nascent products (e.g., glycoprotein), leading to a block in protein synthesis. As can be seen from Figure 6(c), the general trend of metabolites in UDP-N-acetyl-hexosamine pathway is upregulated at 24 h and downregulated at 48 h.

**Figure 7:**
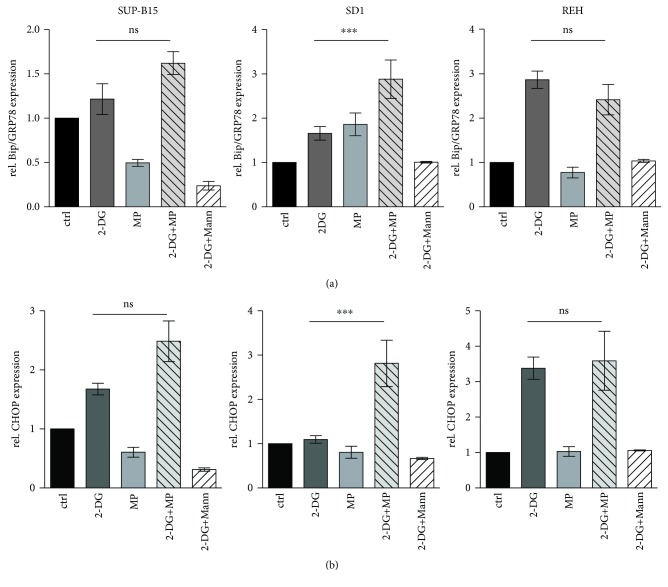
UPR induction by 2-DG in sensitive and resistant ch-ALL cell lines. (a) GRP78/Bip and (b) CHOP mRNA expression was assessed by real-time RT-PCR (SYBR-green) in leukemia cells treated with 2-DG (dark grey bar), MP (light grey bar), 2-DG+MP (grey striped bar), or 2-DG+Mann (white striped bar). Error bars represent the SD of the mean of at least three independent experiments (*n* = 3). *β*-Actin was used as a control to normalize the samples. *p* < 0.05 (ns), ^∗∗∗^*p* ≤ 0.001 using two-way analysis of variance (ANOVA) with Bonferroni's multiple comparison test.

In addition, depending on the cell line used, UDP-N-acetyl-hexosamine levels stay the same or are increased upon treatment with 2-DG, apart from a slight decrease at 6 h. However, treatment with MP results in a general decrease in UDP-N-acetyl-hexosamine, with the exception of REH. The combined treatment shows a more complicated picture, with generally increased levels of the UDP-N-acetyl-hexosamine until 24 h and then falling sharply at 48 h (Figure 6(d)). Together, these results demonstrate the importance of the deregulation of NLG for the biological effects of 2-DG in ch-ALL. It has been described that impaired NLG leads to the accumulation of unfolded proteins within the endoplasmic reticulum (ER) and to the induction of the unfolded protein response (UPR) [24–26]. To investigate whether 2-DG treatment alone or in combination with MP triggers the UPR, leukemia cell lines were exposed to both drugs as single agents or in combination. Moreover, treatments with 2-DG and mannose (2.5 mM) were assessed. The impact of these drugs on the UPR was evaluated by measuring the expression of glucose-regulated protein 78 (GRP78). In addition, the activation of the UPR-mediated apoptotic pathway was investigated after 24 h treatment by measuring the expression of DNA damage-inducible transcript 3 protein (also called CHOP) (Figure 7). 2-DG alone induced an increase in GRP78 expression leading to the activation of the UPR response, in particular in the REH resistant cell type (Figure 7(a)). Surprisingly, in this cell line, the combination of 2-DG and MP did not lead to any additional increment in the expression of GRP78. SUP-B15 and SD1 displayed a significant increase in the levels of GRP78 in cells cotreated with 2-DG and MP, compared to single treatments. Surprisingly, in SD1 cells treated with a single dose of MP (150 *μ*g/ml), the level of GRP78 was increased to the same extent as for the 2-DG single treatment. The activation of UPR-mediated apoptosis via CHOP was evaluated and we were able to show that, for SUP-B15 and SD1, the cotreatment with 2-DG and MP led to a significant increase in the expression of CHOP. In contrast, in REH, the same trend was observed for GRP78, indicating that the treatment with 2-DG alone at 1 mM was sufficient to increase the expression of both genes (GRP78 and CHOP). Moreover, mannose (2.5 mM) cotreatment with 2-DG was able to reverse the effects of the latter drug on GRP78 and CHOP levels in all three leukemia cell lines (Figure 7). These results indicate that in the leukemic cell lines under study, 2-DG induces its biological effects by interfering with NLG leading to UPR induction and subsequent activation of the UPR-mediated apoptotic pathway.

### 3.6. High-Throughput Screen with 365 Protein Kinase Inhibitors Identifies GSK-3 as a Potential Target in Leukemia

**Figure 8:**
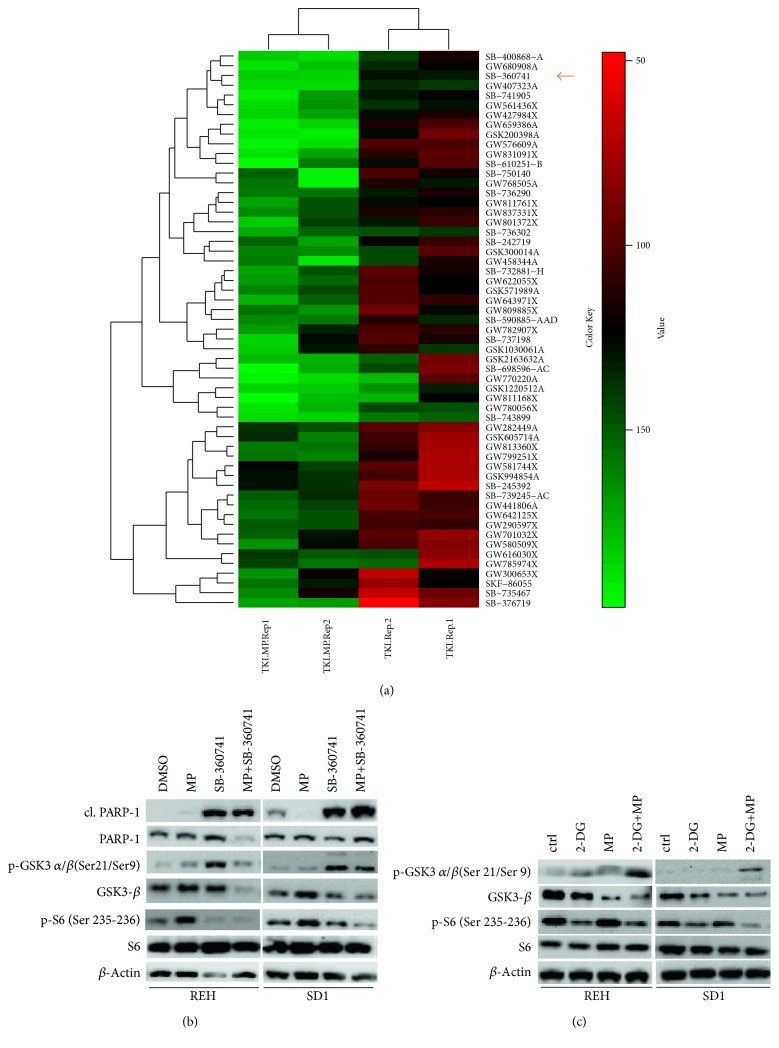
High-throughput screen of protein kinase inhibitors in SD1 and REH leukemia cells. (a) Heat map of REH representing the top candidates from the drug screen upon single and cotreatment with MP. SB-360741, a powerful GSK-3 inhibitor, is indicated by a red arrow. The values shown were calculated as a percentage of the DMSO-treated control. Protein expression profiles of leukemia cell lines, SD1 and REH, upon single or combinatorial treatment with (b) MP and SB-360741or (c) MP and 2-DG.

To better understand the mechanism of action of MP in resistant leukemic cells, we screened 365-protein kinase inhibitors in SD1 and REH cells. The outcome of the screen revealed 57 hit compounds, which acted synergistically with MP. Surprisingly, the majority of those inhibitors targeted kinases involved in metabolism such as p38-mitogen-activated protein kinase (p38/MAPK), insulin-like growth factor-I receptor (IGF-IR), and inhibitor of nuclear factor kappa-B kinase subunit *α* and *β* (IKK-*α*/*β*). Interestingly, among these hit compounds, 14 targeted glycogen synthase kinase-3 (GSK-3). Considering the impact of MP on cell metabolism and the relevance of GSK-3 inhibition in our screen, we decided to investigate the role of this kinase in MP-resistant ch-ALL cells in more detail. We selected the GSK-3 inhibitor SB-360741, a 3-anilino-4-arylmalemide, for further analysis, due to its robust synergistic effects with MP (*F*_syn_ = 0.47 and 0.88 for REH and SD1, resp.) [27]. A significant decrease in REH cell viability was observed after 72 h of treatment (Figure 8(a)). Comparable results were observed in the SD1 cell line (data not shown). In order to investigate the mechanism of cell death induced by MP and SB-360741, we further analyzed the expression levels of several apoptotic markers, which revealed that this process is the main mechanism underlying the observed cytotoxicity. Upon 24 h of single SB-360741 treatment, we observed a slight increment in cells in early apoptosis (Annexin-V-positive cells). However, when SB-360741 was combined with MP, the apoptotic rate increased significantly in comparison to single treatments (Additional file 1: Figure 5). Interestingly, we observed similar biological effects of SB-360741 and 2-DG when coadministrated with MP. We hypothesize a possible link between these two pathways. Therefore, we investigated the effect of 2-DG treatment on GSK-3 activity more in details. Upon single treatment with 2-DG, GSK-3 phosphorylation status remained unchanged, while the combinatorial treatment with MP led to the inhibition of GSK-3 activity via phosphorylation at Ser21 of the *α* isoform. Together, these results reveal the existence of 2-DG- and/or GSK-3-mediated pathway acting synergistically with MP, leading to the induction of cell death and apoptosis in ch-ALL.

## 4. Discussion

Within the last years, a large variety of different solid tumors have been associated with increased metabolic activity [8, 28]. Although there are many examples of solid tumors having altered metabolism, it has been only recently reported that this phenomenon also occurs in hematological malignancies [21, 22, 29, 30]. Therefore, targeting the metabolic pathways could represent an innovative approach to sensitize leukemic cells to chemotherapeutic drugs and potentially lead to a better patient outcome [31, 32]. One of the most important prognostic factors in ch-ALL is the response to the GCs treatment. Thus, strategies aiming to reverse GCs resistance could improve the survival of children suffering from leukemia. Recent data investigating the mechanisms involved in MP resistance showed a tight correlation between rapid leukemic growth and a high glycolytic ratio [5]. Therefore, it is of interest to further analyze the impact of glycolysis inhibitors in leukemia, since several in vitro and in vivo studies have demonstrated their efficacy as anticancer drugs [33]. Moreover, some of these inhibitors have been already selected as candidates for clinical trials [21, 29, 34, 35]. In our study, the glucose analog 2-DG was selected principally because of its strong activity in depleting the production of ATP at the first limiting step of glycolysis. The investigation of the impact of 2-DG, as an antileukemic agent led us to the hypothesis that leukemia cells, like solid tumors, might have an altered metabolism, a phenomenon termed as “aerobic glycolysis” [36]. Indeed, the inhibition of the glucose consumption by 2-DG was paralleled by a decrease in cell viability and enhanced apoptosis in our in vitro leukemia model. However, a general downregulation of the expression of genes involved in glycolysis was revealed by cDNA microarray data analysis performed on a cohort of ch-ALL patients. A remarkable decrease was particularly observed in the expression of HK isoforms responsible for the biological effect of 2-DG. Furthermore, the cytotoxic effects of 2-DG in cancer appears to be higher under hypoxic conditions, or in cells presenting mitochondrial defects [37, 38]. Since leukemia cells have the capacity to circulate freely through normoxic and hypoxic environments, it was of importance to investigate the different mechanisms involved in 2-DG-induced cytotoxicity. Our data show that the glucose analog acts in an HKII-independent manner to reduce cell growth in leukemia. In support of our findings, several reports have suggested a more complex mechanism of action of 2-DG [12, 39, 40]. In this context, we were able to show that a significant fraction of the 2-DG-mediated cell death can be rescued by coadministration of exogenous mannose. These data suggest that the observed antileukemic effects of 2-DG potentially involve NLG. By interfering with this process and consequently with protein folding, 2-DG promotes ER stress leading to UPR induction and protein synthesis inhibition [41, 42]. In addition, prolonged ER stress triggers an irreversible UPR-mediated apoptotic response [43]. The deregulation of NLG via 2-DG might serve as a sensor linking metabolism with the activation of oncogenic pathways. In line with this, a screen of 365 protein kinase inhibitors performed in two leukemia cell lines revealed that most of the kinases synergistically acting with MP were involved in metabolism. The selected kinase inhibitor targeting GSK-3 (SB-360741) was able to induce the same biological effects as 2-DG in combination with MP. GSK-3 is known to regulate apoptosis and proliferation in cancer and its role appears to be distinct, not only between physiological and pathological conditions but also depending on cell type and tissue context [44–46]. Moreover, in neuroblastoma, 2-DG exposure triggered an energy depletion in a GSK-3-mediated manner resulting in attenuation of the mitochondrial biogenesis, a particular form of stress adaptation [47]. In leukemia cell lines, we were unable to show a direct regulation of GSK-3 by 2-DG. Nevertheless, the combinatorial treatment of the glucose analog with MP highly affected the activity of the kinase. In conclusion, this study provides new insights into the intimate connection between GCs resistance and altered metabolism in ch-ALL. Therefore, it will be of a great interest to investigate alternative roles of 2-DG in a wider metabolic context, besides its function as a glucose analog. In addition, investigating of the role of GSK-3 in leukemia metabolism will provide further essential knowledge about the mechanisms underlying MP resistance. This research work provides evidence of the intimate connection between GCs resistance and altered metabolism in ch-ALL. Therefore, the current and the future challenge in the field of cancer metabolism is to dissect these complex metabolic changes and interpret them as a result of global metabolic interactions.

## Supplementary Material

S1: Scatter diagram of regression analysis. S2: Expression level of genes involved in metabolism in leukemia cell lines. S3: Cell viability of ch-ALL cells in normoxia and hypoxia condition. S4: HK expression in leukemia cell lines. S5: Inhibition of GSK3 alpha with SB-360741.
