# Supplementary figures and images for: 2-Deoxy-D-glucose Restore Glucocorticoid Sensitivity in Acute Lymphoblastic Leukemia via Modification of N-Linked Glycosylation in an Oxygen Tension-Independent Manner
Source: Oxid Med Cell Longev. 2017 Jul 26;2017:2487297. doi: 10.1155/2017/2487297 (PMC5549481; doi:10.1155/2017/2487297)

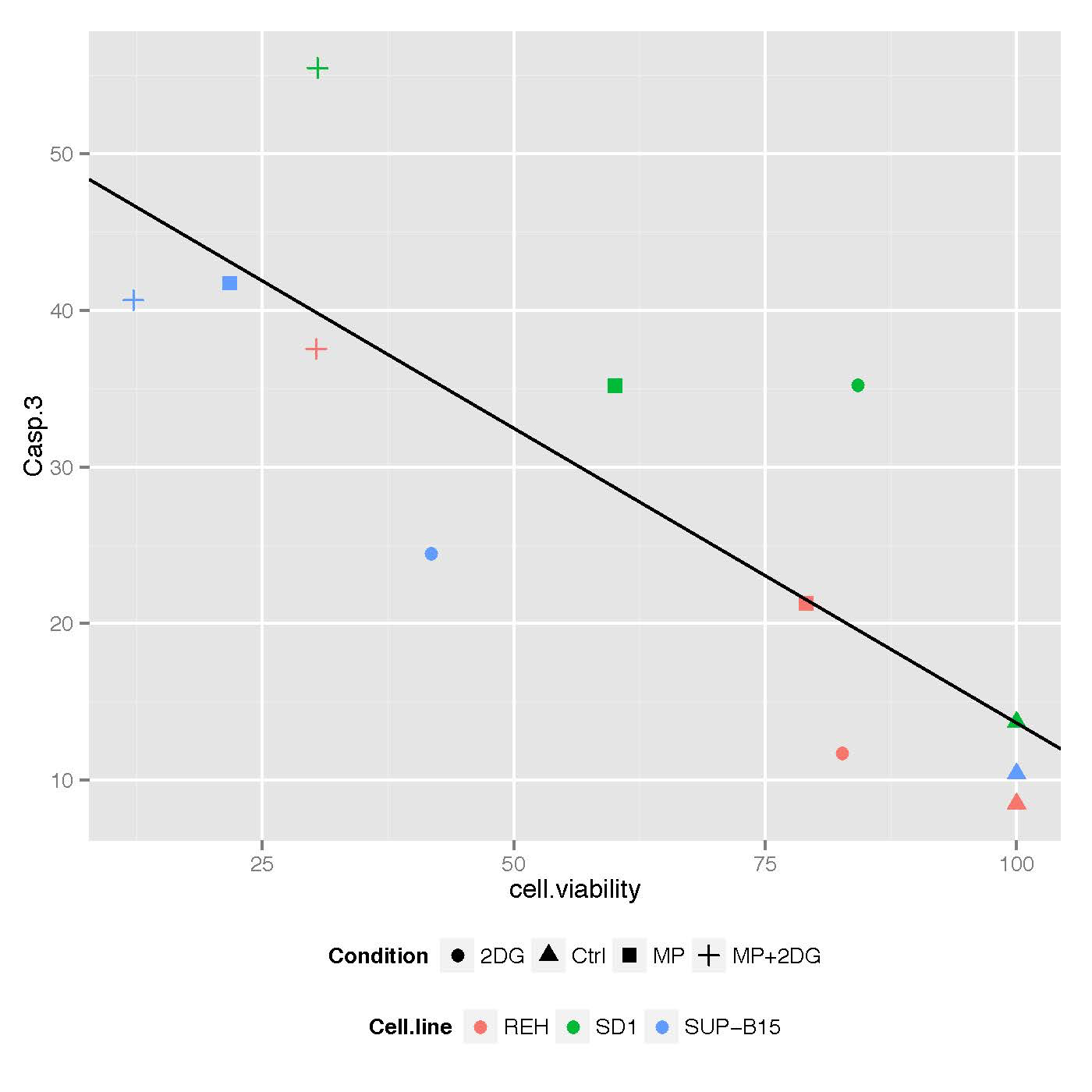

Supplement: Supplementary file 1 — S1: Scatter diagram of regression analysis. S2: Expression level of genes involved in metabolism in leukemia cell lines. S3: Cell viability of ch-ALL cells in normoxia and hypoxia condition. S4: HK expression in leukemia cell lines. S5: Inhibition of GSK3 alpha with SB-360741. [file 2487297.f1.docx]

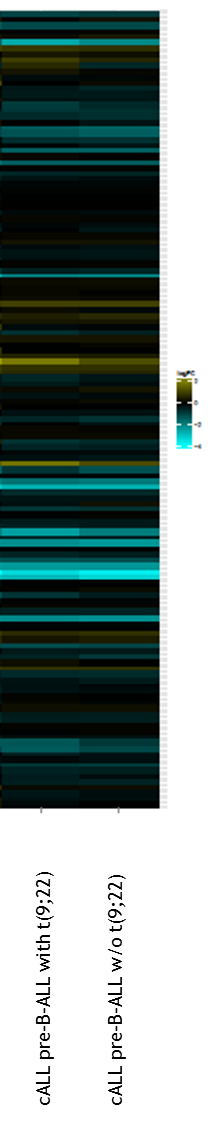

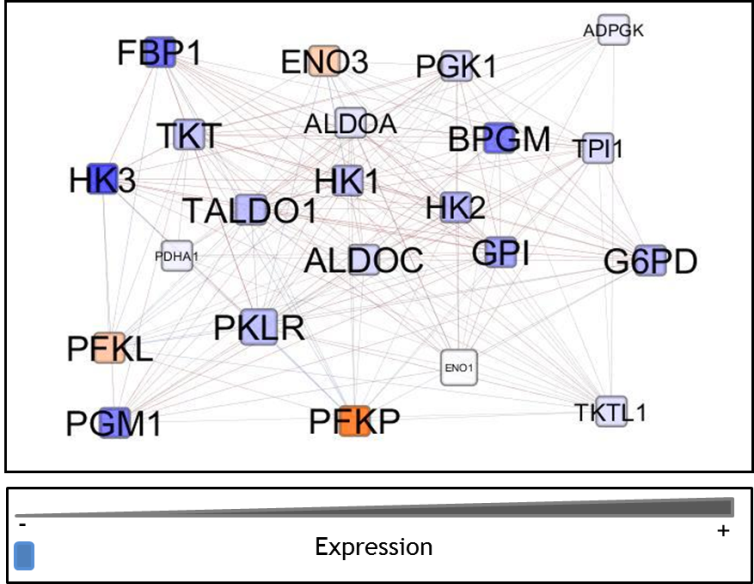

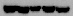


*B-actin*

*41bMI*

*FIN COS*

*SUP-B15*

*SD1*

*REH*

*HK-3*

*HK-2*


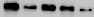


*HK-1*

**A.**

**C.**

**B.**

Supplement: Supplementary file 2 [file 2487297.f2.docx]
