# Supplementary material for: 2-Deoxy-D-glucose Restore Glucocorticoid Sensitivity in Acute Lymphoblastic Leukemia via Modification of N-Linked Glycosylation in an Oxygen Tension-Independent Manner
Source: Oxid Med Cell Longev. 2017 Jul 26;2017:2487297. doi: 10.1155/2017/2487297 (PMC5549481; doi:10.1155/2017/2487297)
Supplement: Supplementary file 4 [file 2487297.f4.docx]

**A.**

**B.**
