# Supplementary material for: 2-Deoxy-D-glucose Restore Glucocorticoid Sensitivity in Acute Lymphoblastic Leukemia via Modification of N-Linked Glycosylation in an Oxygen Tension-Independent Manner
Source: Oxid Med Cell Longev. 2017 Jul 26;2017:2487297. doi: 10.1155/2017/2487297 (PMC5549481; doi:10.1155/2017/2487297)
Supplement: Supplementary file 3 [file 2487297.f3.docx]

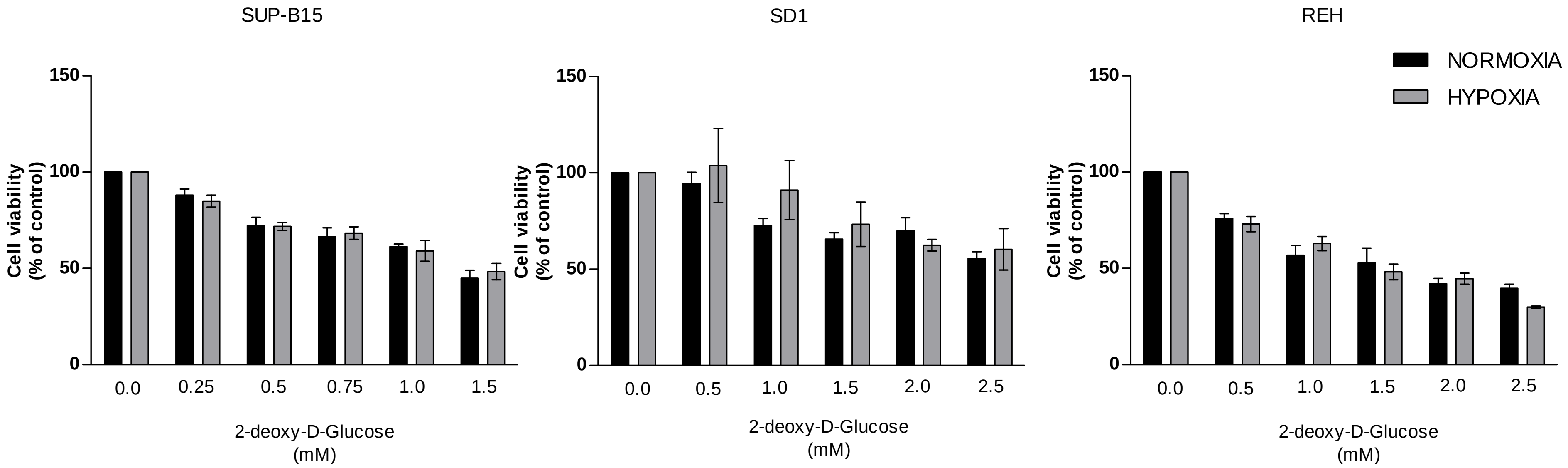
SUP-B15
SD1
REH
NORMOXIA
HYPOXIA
Cell viability
(% of control)
150
100
50
0
0.0
0.25
0.5
0.75
1.0
1.5
2.0
2.5
2-deoxy-D-Glucose
(mM)
