# Supplementary material for: 2-Deoxy-D-glucose Restore Glucocorticoid Sensitivity in Acute Lymphoblastic Leukemia via Modification of N-Linked Glycosylation in an Oxygen Tension-Independent Manner
Source: Oxid Med Cell Longev. 2017 Jul 26;2017:2487297. doi: 10.1155/2017/2487297 (PMC5549481; doi:10.1155/2017/2487297)
Supplement: Supplementary file 4 [file 2487297.f5.docx]

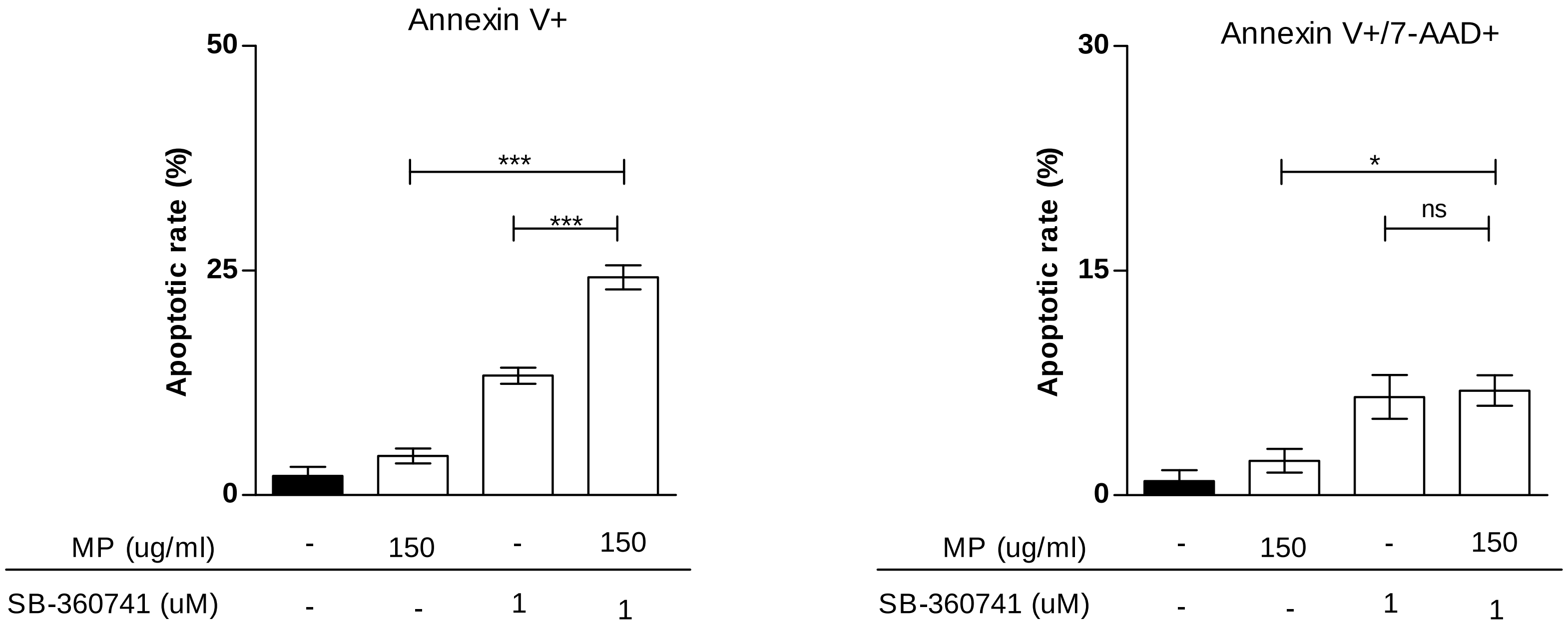

Annexin V+
Apoptotic rate (%)
50
25
0
***
***
MP (ug/ml)
-
150
-
150
SB-360741 (uM)
-
-
1
1
Annexin V+/7-AAD+
Apoptotic rate (%)
30
15
0
*
ns
MP (ug/ml)
-
150
-
150
SB-360741 (uM)
-
-
1
1
